# Theranostic of orthotopic gliomas by core-shell structured nanoplatforms

**DOI:** 10.1038/s41377-022-00852-2

**Published:** 2022-05-20

**Authors:** Luís D. Carlos

**Affiliations:** Phantom-G, CICECO-Aveiro Institute of Materials, Physics Department, University of Aveiro, 3810-193 Aveiro, Portugal

**Keywords:** Nanoparticles, Biophotonics

## Abstract

Smart designed core-shell nanostructures formed by a YVO_4_: Nd^3+^ nanoparticle as the core, the sonosensitizer hematoporphyrinmonomethyl ether as the carrier, and MnO_2_ nanosheets as the shell demonstrate bimodal imaging and highly efficient sonodynamic therapy of orthotopic gliomas.

Glioma is a tumor originating from glial cells that accounts for ca. 75% of malignant primary brain tumors in adults. Current treatment includes surgery, radiation therapy, and chemotherapy but despite the tremendous efforts made in the past decade, the 5-year overall survival rate is still low^1^ mainly because the majority of drugs and contrast agents cannot pass through the blood-brain barrier (BBB)^2,3^. Consequently, it is extremely important to develop new treatments for gliomas.

Among the external-stimuli-triggered noninvasive therapies, sonodynamic therapy (SDT)^4^ is an emerging and promising approach offering the possibility of in situ eradicating tumors, including in the brain^5^. It involves the combination of low-intensity ultrasound as the irradiation source with a chemotherapeutic agent (sonosensitizer) to generate reactive oxygen species and its main advantages are the high tissue-penetrating depth, no phototoxicity, controllability, and safety^5,6^. As SDT consumes oxygen in the tumor microenvironment, increasing the oxygen supply to the tumor is mandatory to alleviate hypoxia and improving the efficacy of the technique^5^. In recent years, MnO_2_ nanomaterials have been recognized as a promising type of biodegradable tumor microenvironment-responsive O_2_ producers and magnetic resonance imaging (MRI) contrast agents^7^. In fact, MnO_2_ nanostructures can trigger the decomposition of H_2_O_2_ existing in the tumor microenvironment into water and oxygen relieving tumor hypoxia and its decomposition (by reaction with either H+ or glutathione) generates harmless water-soluble Mn^2+^ ions that can significantly enhance T1-weighted MRI contrast for tumor-specific imaging and detection^7^.

Fluorescence imaging in the second near-infrared window (NIR-II, 1000–1700 nm) has come up recently as one of the fastest developing and most widely used imaging technologies for biomedical applications due to its unique characteristics, including rapid feedback, multiple signal acquisition capability, high sensitivity, and spatial resolution, low tissue absorption and scattering, and the absence of ionizing radiation^8,9^. Moreover, there is an emerging interest in the integration of distinct imaging agents, namely NIR-II and MRI ones^10^, into multifunctional nanoparticles to exploit the potential of combining the advantages and minimizing the disadvantages of different imaging modalities^11^. Among the scrutinized NIR-II probes, trivalent lanthanide-doped nanoparticles are recognized as promising for through-skull targeted imaging^12^ thanks to their high emission quantum yield, narrow bandwidth, long-lived emission, large Stokes shifts, and ligand-dependent luminescence^13^.

Now, writing in this issue of *Light: Science & Applications*, Zhijia Lv and colleagues at the Changchun Institute of Applied Chemistry, University of Science and Technology of China, Ganjiang Innovation Academy, Tsinghua University, The First and the Second Hospitals of Jilin University in China, and the National University of Singapore in Singapore, design and construct a leading-edge NIR-II/MRI bimodal core-shell nanotheranostic agent for efficient SDT of orthotopic gliomas^14^. The smart-designed nanostructure consists of a YVO_4_: 25% Nd^3+^ core with the hematoporphyrinmonomethyl ether sonosensitizer loaded onto its surface and a MnO_2_ shell functionalized with lactoferrin (Fig. 1). The core enables Nd^3+^ NIR-II imaging of blood vessels and orthotopic glioma whereas the shell generates O_2_ and releases Mn^2+^ ions in the tumor environment upon ultrasound irradiation to enhance the treatment effect of SDT, enabling concomitantly in situ T1-weighted MRI. The successful crossing of the BBB to target gliomas is warranted by the shell functionalization with lactoferrin.

**Fig. 1:**
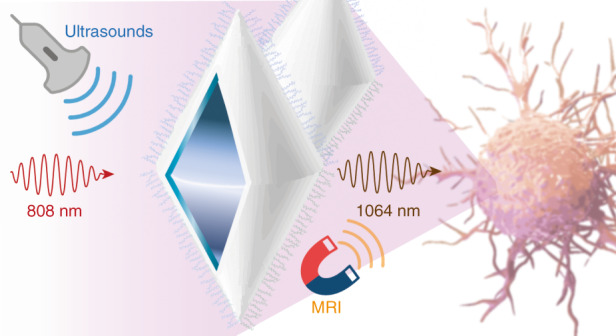
Schematic illustration of the bimodal core-shell nanostructures designed by Lv et al.^14^ to overpass the BBB and target orthotopic gliomas. NIR-II and MRI are induced by simultaneous ultrasound and 808 nm irradiation

With the presented work, Zhijia Lv and co-authors demonstrate in vitro and in vivo (on rats) a successful approach to inhibiting the growth of orthotopic gliomas by enhanced non-invasive SDT. While the potential of Mn^2+^-doped nanoparticles and nanocomposites for SDT has already been pointed out^12,15^, the *lego principle* behind the smart design of the reported core-shell nanostructure could be a significant contribution to the development of multifunctional agents for bioimaging and SDT therapy and, hence, new non-invasive treatments for gliomas. Future prospects might include the optimization of the designed core-shell structure incorporating, for example, brighter NIR-II emitters and other bioimaging agents, e.g., positron emission tomography(PET)/single photon emission computed tomography (SPECT) radiotracers and luminescent nanothermometers.
